# Measuring the impact of the COVID-19 epidemic on university resumption and suggestions for countermeasures

**DOI:** 10.3389/fpubh.2022.1037818

**Published:** 2022-12-19

**Authors:** Shi Yin, Lijun Ma, Tong Dong, Ying Wang

**Affiliations:** ^1^College of Economics and Management, Hebei Agricultural University, Baoding, China; ^2^College of Land and Resources, Hebei Agricultural University, Baoding, China

**Keywords:** resumption of universities, risk prevention and control, comprehensive risk assessment, COVID-19, fuzzy statistical model

## Abstract

**Background:**

During the COVID-19 pandemic, universities around the world had to find a balance between the need to resume classes and prevent the spread of the virus by ensuring the health of students. The purpose of our study was to effectively assess the overall risk of universities reopening during the COVID-19 epidemic.

**Design and methods:**

Using the pressure–state–response model, we designed a risk evaluation method from a disaster management perspective. First, we performed a literature review to find the main factors affecting the virus spread. Second, we used the pressure–state–response to represent how the considered hazards acts and interacts before grouping them as disaster and vulnerability factors. Third, we assigned to all factors a risk function ranging from 1 to 4. Fourth, we modeled the risk indexes of disaster and of system vulnerability through simple and appropriate weights and combined them in an overall risk for the university resumption. Finally, we showed how the method works by evaluating the reopening of the Hebei Province University in 2022 and highlighted the resulting advice for reducing related risks.

**Results:**

Our model included 20 risk factors, six representing exogenous hazards (disaster factors) that university can only monitor and 14 related to system vulnerability that can also control. Disaster factors included epidemic risk level of students' residence and the school's location, means of transportation back to school, size of the university population, the number of migrants on and off campus and express carrier infection. Vulnerability factors included student behaviors, routine campus activities and all the other actions the university can take to control the virus spread. The university of Baoding city (Hebei Province) showed a disaster risk of 1.880 and a vulnerability of 1.666 which combined provided a low risk of school resumption.

**Conclusion:**

Our study judged the risks involved in resuming school and put forward specific countermeasures for reducing the risk levels. This not only protects public health security but also has some practical implications for improving the evaluation and rational decision-making abilities of all parties.

## 1. Introduction

The novel coronavirus disease 2019 (COVID-19) is an ongoing pandemic that has evolved into a global crisis and has seriously challenged the development process of human society (1). The COVID-19 pandemic is a major worldwide public health emergency that spreads fast over a wide range of locations and is difficult to prevent and control; however, thanks to the concerted efforts of people around the world and to a global vaccination campaign, the epidemic prevention and control situation has continued to improve, and the order of work and life was quickly restored (2). Despite this, the appearance of virus variants with higher infectivity makes the pandemic still not under effective control (3). Tertiary education Institutions play a key role in assuming the functions of higher education and are also an important public field to manage emergencies (4). In the COVID-19 context, many colleges and universities have issued plans to reopen their school even if before the pandemic showed high incidences of respiratory infectious diseases. While resuming in-person teaching, the back-to-school activities mean further battles for epidemic prevention and control for several reasons. First, colleges and universities need to be effective regarding the prevention, monitoring, and management of public health emergencies. Measures taken by colleges and universities often lag behind the development speed of the crisis, and the phenomenon of post-management rather than prevention always exists. For example, emergency warning mechanisms in Chinese universities have been ineffective due to the uncertainty of public health emergencies and technology defects. Second, the ability to cope with and guide public opinion in a public health crisis is a considerable aspect of university governance modernization, especially in the Internet era. As a relatively closed social cluster, the spread of rumors and false information in a university can easily cause panic among teachers and students, creating additional considerations for governing public health emergencies (5). Social media has rapidly developed, and society has entered an information age. Young people use social media much more frequently than other age groups, making it more difficult for universities, which are mainly composed of young college students, to curb false information. Third, the education system has entered the mobile war stage of epidemic prevention and control, and risk factors have become more complex and changeable (6). The full resumption of education in colleges and universities has brought about a larger range and scale of personnel mobility. The activities and management issues of overseas students have brought new risks to epidemic prevention and control in schools, which has changed from positional to mobile anti-disease warfare. In addition, as epidemic prevention and control has entered the normalization stage, various associated problems emerged, such as stress responses, anxiety, and other psychological problems; livelihood issues, such as entering schools and resuming employment; and teaching management issues, such as the connection between online and offline teaching. In China, the problem has been investigated with different approaches and several solutions have been proposed. Yang (7) constructed a risk assessment system for school respiratory infectious disease outbreaks from four perspectives: possibility, vulnerability, severity, and countermeasures. Liu and Zhang (8) discussed applying a risk assessment of the overall smart campus framework in terms of risk identification, assessment, disposal, and control to form a set of network security risk assessment methods that can be widely applied to the current overall smart campus frameworks. Ding and Li (9) proposed a Delphi method and AHP method combined with Borda ordinal value method to study the risks after returning to school under the COVID-19 epidemic. Wang et al. (10) discussed a risk assessment method for reopening universities that can evaluate the comprehensive risks of resuming education during the COVID-19 epidemic and assist universities in making organizational decisions for reopening. Although the latter study analyzed the interaction mechanisms of various factors based on pressure–state–response model and established a comprehensive risk assessment index system for COVID-19 outbreaks in colleges and universities, important factors involved during education resumption are missing. Starting from the previous results, the aim of this study is to introduce a comprehensive index to measure the risk of virus spread during university resumption and to take a university as our research object.

## 2. Methods

The proposed risk evaluation tool is designed from a disaster management perspective and is based on the pressure–state–response model. First, we have selected the main factors affecting the virus spread that are the most considered in literature. The selection of these factors is based on the five principles of significance, operability, practicability, relevance and concreteness of the index system, PSR model, and many literature (2–22). Second, we used the pressure–state–response to represent how those hazards acts and interacts before grouping them in disaster and vulnerability factors. Levels of categorical factors (such as means of transport) were ranked from lowly to highly dangerous and recoded with values corresponding to the risk rank (such as Self-driving = 1, Taxi = 2, Train/R = 3, Other = 4). Third, according to Chinese 3-levels territorial epidemic risk (high, medium, and low),^1^ to simplify the computational process we used step functions to assign risks to factors values ranging from 1 to 4. The choices of those functions are based on the experience of the risk assessment expert group composed of experts in various fields such as medicine, education and emergency management. Fourth, we modeled the risk indexes of disaster and of system vulnerability as weighted mean of related indexes and combined them in and overall risk for the university resumption. Finally, we showed how the method works by evaluating the reopening of the Hebei Province University in 2022 and highlighted the resulting advice for reducing related risks.

### 2.1. PSR model

**Figure 1:**
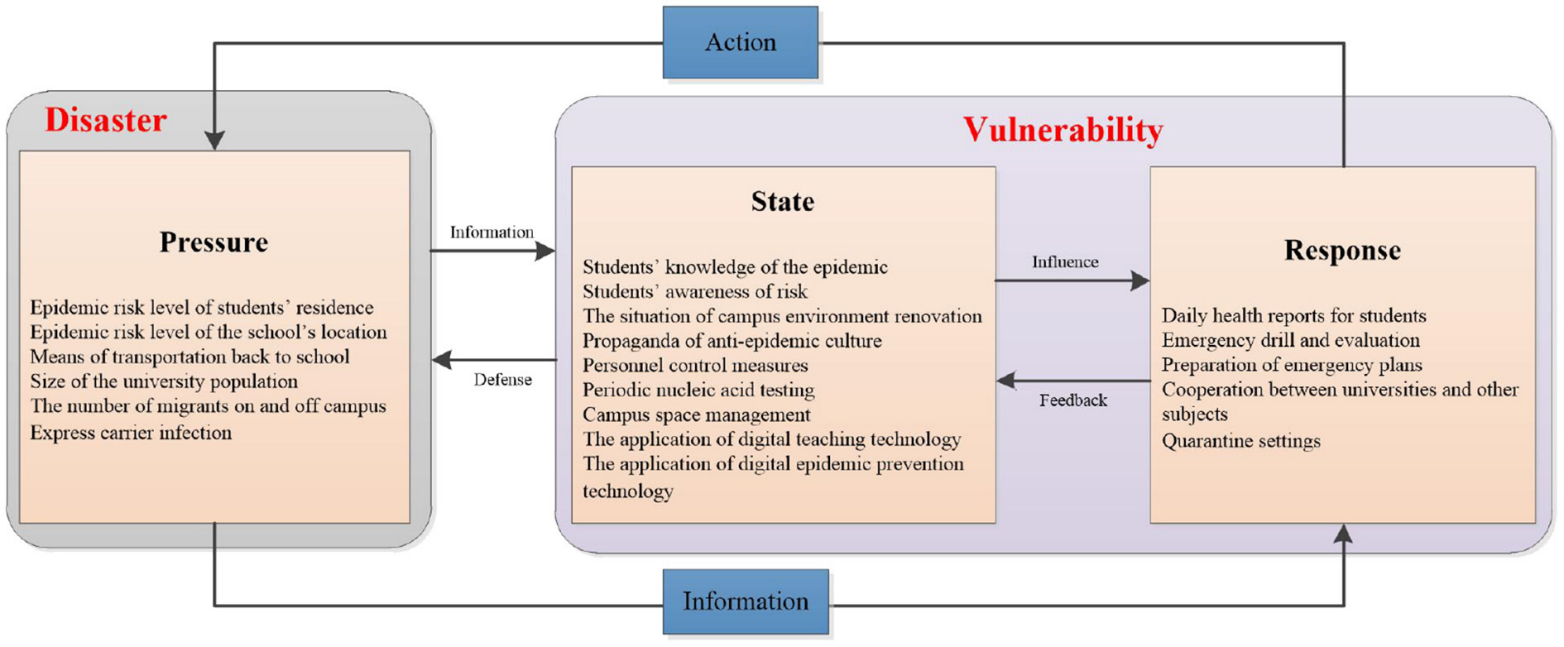
PSR mechanism model of epidemic risk situation in universities from a disaster management perspective.

The PSR framework models the chain of causal links between a system working to maintain a state, and exogenous forces working to change it. Formally, it is an interconnected conceptual structure consisting of three parts (11): pressure, which represents the process of adverse effects generated from the system interference and coercion (12); state, which represents the current state of the system under external pressure (13); response, which represents the feedback process of the system in response to external pressure (14). Even if domestic scholars generally use this theory to explain phenomena in the fields of taxation and ecological and environmental protection (15), it can also reflect the dynamic processes and internal logic of university environments. All the factors that affect the epidemiological risks associated with university resumption and all the involved subjects interact and influence each other in a dynamic balance (Figure 1). The epidemic situation before students return to school, the public transportation they take on the way back to school, and the flow of people inside and outside the school after they return increase the risk of epidemic transmission and together form the pressure system. Information released by the pressure system allow involved subjects to take countermeasures. The status system includes student behaviors and routine campus activities, such as raising students' risk awareness, adopting online education, distance learning and strengthening campus space management to reduce the risk of the virus spreading. The response system includes all the measures a university can take that do not strictly concern the routine campus activity, such as emergency plans, drills, and assessments; the establishment of isolation sites; and the development of response systems to deal with outbreaks. The epidemic prevention and control status quo in colleges and universities will directly affect the response measure effectiveness and timelines, and improvements in response capacity will feed back to the status system and improve the prevention and control status quo.

Methods for detecting and measuring selected risk factors are summarized in Supplementary material 1 and described in the following sections.

#### 2.1.1. Indicators of pressure system

Since the movement of people inside and outside the school will increase the risk of the epidemic spreading, as indicators of the Pressure System we selected: epidemic risk level of students' residence (P_1_) (13); epidemic risk level of the school's location (P_2_) (6); means of transportation back to school (P_3_) (6); size of the university population (P_4_) (16); the number of migrants on and off campus (P_5_) (16); and express carrier infection (P_6_) (17). According to national classification of territorial risk (see text footnote 1), we assigned to each level of P_1_ and P_2_ risks equal to 1.5 (low), 2.5 (medium) or 3.5 (dangerous). Risk of P_3_ was assessed as the weighted mean of risks of the 4 most used vehicles such as Self-driving, Taxi, Train/R, Other. Means of transportation were ranked by the expected number of close contacts, then we assigned equidistant risk values (Self-driving = 1, Taxi = 2, Train/R = 3, Other = 4) weighted with fraction of users. Risk values of 1, 2, 3, 4 were assigned to P_4_, P_5_, and P_6_ as follows: if the number (*n*) of students returning to school/1,000 was *n* < 0.5, 0.5 ≤ *n* < 1, 1 ≤ *n* < 2, 2 ≤ *n* respectively; if the percentage (p) of students entering and leaving the school was p < 5%, 5% ≤ p < 15%, 15% ≤ p < 25%, p ≤ 25% respectively; if the percentage (p) of express deliveries from medium and dangerous risk places was p < 5%, 5% ≤ p < 15%, 15% ≤ p < 25%, p ≤ 25% respectively.

#### 2.1.2. Indicators of state system

We analyzed the state system from the perspective of individual students, the campus environment, management measures, and the use of digital technology. As indicators we selected: students' knowledge of the epidemic (S_1_) (9); students' awareness of risk (S_2_) (10); the situation of campus environment renovation (S_3_) (18); propaganda of anti-epidemic culture (S_4_) (19); personnel control measures (S_5_) (20); periodic nucleic acid testing (S_6_) (21); campus space management (S_7_) (18); the application of digital teaching technology (S_8_) (22); and the application of digital epidemic prevention technology (S_9_) (6). Risk of S_1_ was assessed by assign risk values to the students' knowledge level of epidemic prevention and control (*X*) of 4 – 3(*X* – 8)/(10 – 8) and 4 if 8 ≤ *X* and *X* < 8, respectively. Risk values of 1, 2, 3, 4 were assigned to S_2_–S_9_ as follows: if the risk awareness of the students observed in daily behavior was very strong, strong, general, and weak, respectively; if the campus environment renovation (cleaned up campus health dead spots; disinfected public places; equipped with disinfectants and hand sanitizer) was completed (three items), almost completed (two items), started (one item), and not started (0 item), respectively; if the percentage (p) of promoted prevention and control culture was 50% ≤ p, 30% ≤ p < 50%, 15% ≤ p < 30%, p < 15% respectively; if the organizational framework of colleges and universities was met the requirements, slightly defective, major defects, failure to formulate a reasonable organizational institutional framework, respectively; if the frequency (*p*) of nucleic acid testing was once a day, three times a week, twice a week, once a week respectively; if the distance (*d*) in meters (m) among the students was 2 m ≤ *d*, 1.5 m ≤ *d* < 2 m, 1 m ≤ *d* < 1.5 m, *d* < 1 m respectively; if the percentage (p) of the satisfaction of teachers and students with online teaching was 95% ≤ p, 90% ≤ p < 95%, 80% ≤ p < 90%, p < 80% respectively; if the percentage (p) of student nucleic acid testing system registration was 95% ≤ p, 90% ≤ p < 95%, 80% ≤ p < 90%, p < 80% respectively.

#### 2.1.3. Indicators of response system

According to the mechanisms of the discovery, initiation, and control of emergency responses, as indicators of the Response System we selected: daily health reports for students (R_1_) (6); emergency drill and evaluation (R_2_) (19); preparation of emergency plans (R_3_) (2); cooperation between universities and other subjects (R_4_) (6); and quarantine settings (R_5_) (2). Risk of values of 1, 2, 3, 4 were assigned to R_1_–R_5_ as follows: if the health monitoring days per student in the data system (d) was 14 ≤ d, 7 ≤ d < 14, 0 ≤ d < 7, unestablished system respectively; if the school epidemic drill situation was conducted as emergency drills for evaluation and made improvements based on evaluation comments, emergency drills and assessments but did not improve all assessments, emergency drills but did not conduct drill evaluations or unconducted respectively; if the emergency response plan was well-prepared contingency plans for various emergencies, comparatively perfect preparation of various contingency plans, inadequate preparation of contingency plans for various emergencies, failure to prepare for various emergencies respectively; if the university collaborated with three, two, one or no organizations respectively; if the isolation area and the personnel on duty was evaluated as adequate quarantine areas and staff on full-day duty, inadequate quarantine areas and staff on full-day duty, adequate quarantine areas and staff not on duty all day, inadequate quarantine areas and staff not on duty all day respectively.

### 2.2. Overall risk evaluation

From the previous three indicators, we obtained an overall risk evaluation by using a disaster management perspective. In the specific, the risk degree (*R*) of reopening a university was evaluated through the cartesian product of disaster (*H*) and vulnerability (*V*) factors (10).


(1) $$\begin{array}{l} R=H\times V \end{array}$$

where *H* reflects the pressure subsystem and *V* both the state and response subsystems of the related PSR model (Figure 1). Like in natural disasters, the Equation (1) is suitable to represent the levels of risks related to the virus spread. Indeed, at the onset of outbreaks, interventions on hazards included in *H* may not be rapid enough and differences in the level of epidemic are determined by the capacity to reduce students' vulnerability. Furthermore, to consider the specific universities conditions we introduced index weight settings to adapt to local conditions. The proposed index method first needs to assess *H* and *V* as follows


(2) $$\begin{array}{l} \{\begin{array}{l} H=\sum_{i=1}^{6}p_{i}w_{i}\\ V=W_{S}\sum_{j=1}^{9}s_{j}w_{j}+W_{R}\sum_{l=1}^{5}r_{l}w_{l} \end{array} \end{array}$$

where *p*_*i*_, *s*_*j*_, *r*_*l*_ and *w*_*i*_, *w*_*j*_, *w*_*l*_ represent values and weights of each indicator of pressure (*i* = 1, 2, ⋯   , 6), state (*j* = 1, 2, ⋯   , 9), and response (*l* = 1, 2, ⋯   , 5), respectively. *W*_*S*_ and *W*_*R*_ are the weights of the state and response systems, respectively, which represent the vulnerability of the students to the pandemic. To simplify the complexity of the evaluation system, we used the improved order relation method to determine the weights and to satisfy the weak consistency of the indicator (23).

Risk factors can be ranked from the most to the less important (*C*_1_, *C*_2_..., *C*_m_) by associating to them a corresponding system of non-increasing weights (*w*_1_* ≥ *w*_2_* ≥ ... ≥ *w*_m_*) with $\sum_{i=1}^{m}w_{i}^{*}=1$. By using *C*_i_ to represent the indicators in the subsystem *P*, *S* and *R*, the weight calculation method of each indicator was as follows:

① Experts judged the influence importance of a subsystem's risk value according to each indicator in the subsystem and provided the weight order, which was denoted as


(3) $$\begin{array}{l} w_{\text{1}}\text{*}\geq w_{\text{2}}\text{*}\geq ...\geq w_{m}\text{*} \end{array}$$

② We compared the sorted weight of indicators *C*_*i*−1_($w_{i-1}^{*}$) and *C*_*i*_($w_{i-1}^{*}$), which were denoted using the following formula:


(4) $$\begin{array}{l} r_{i}=\frac{w_{i-1}^{*}}{w_{i}^{*}},i=2,3,\ldots ,m \end{array}$$

For the value of *r*_*i*_, please refer to Table 1 (23).

**Table 1:** Value reference for *r*_*i*_ .

$w_{i}^{*}$	**Instruction**
1.0	$w_{i-1}^{*}$ and $w_{i}^{*}$ have the same contribution
1.2	$w_{i-1}^{*}$ contributes a little more than $w_{i}^{*}$
1.4	$w_{i-1}^{*}$ makes a bigger contribution than $w_{i}^{*}$
1.6	$w_{i-1}^{*}$ has a stronger contribution than $w_{i}^{*}$
1.8	$w_{i-1}^{*}$ definitely contributes more than $w_{i}^{*}$

③ Weight $w_{m}^{*}$ and $w_{i}^{*}$ were calculated one after the other as follows:


(5) $$\begin{array}{l} w_{m}^{*}={\left(1+\sum_{i=2}^{m}\prod_{k=i}^{m}r_{k}\right)}^{-1},\;w_{i-1}^{*}=w_{m}^{*}\prod_{k=i}^{m}r_{k}\;i=2,\cdots \;,m. \end{array}$$

Finally, we used the risk matrix in Zhao and Wang (24) to determine the comprehensive risk level of the epidemic situation during university resumption, and the evaluation results were represented using D (dangerous), M (moderate), and L (low). Table 2 shows the risk matrix of the epidemic situation during university resumption. Risk level D indicates that students' return to school is unacceptable, and the school should immediately stop the return process and make corrections. M means that it is not expected to happen, and management decisions are made to prevent the development of risks. L means it is acceptable, and the risk control measures should be improved accordingly.

**Table 2:** Epidemic risk matrix of resumption in universities.

	***H***		
*>**V***	**(1,2]**	**(2,3]**	**(3,4]**
(1,2]	*L*	*L*	*M*
(2,3]	*L*	*M*	*D*
(3,4]	*M*	*D*	*D*

### 2.3. Case study

We used the university of Baoding city, Hebei Province, as case study. With permission from the Department of Education, the school resumed all in-person activities (under closed management) in February 2022. Since March 14, 2022, online teaching was promptly adopted, (with teachers teaching online at home and students choosing quiet places) because of outbreaks in all of China's provinces. Data related to the risk indicator factors were collected as follows. Risk levels issued by the regional Health Commission were used to measure P_1_ and P_2_. The university has performed a survey of returning students through questionnaire to measure P_3_, P_4_, and P_5_. We asked that express deliveries station fill out the online shared form questionnaire to measure P_6_. We randomly selected 100 students to conduct an epidemic knowledge questionnaire to collect data related to factors S_1_ and S_8_. We asked that the monitor of each class fills out the online shared form questionnaire to collect data related to factor S_2_. We asked that the head of epidemic control checks the item and fills out the online shared form questionnaire to collect data related to factors S_3_, S_6_, S_7_, and S_9_. We asked that the publicity department of the school count the number and methods of anti-epidemic activities and fills in questionnaires to collect data related to factor S_4_. Risk level can be determined by the risk assessment expert group composed of experts in various fields such as medicine, education and emergency management to collect data related to factor S_5_. We collected data related to factor R_1_ from the database of the epidemic prevention and control department. We asked that the head of epidemic control checks the item and fills out the online shared form questionnaire to collect data related to factors R_2_, R_3_, and R_4_. We collected data related to factor R_5_ from the duty record registration form.

## 3. Results

According to the case study, the percent of high risk level, medium risk level and low risk level of students' residence (P_1_) were 6.71, 8.03, and 85.26%, respectively. The regional Health Commission thought that epidemic risk level of the school's location (P_2_) is low risk level. The ways that students returned to school (P_3_) were as follows: 8.6% by car, 64.3% by train (high-speed rail), 6.8% by taxi, and 20.3% by other means. The size of the university population (P_4_) is 1,850. The number of migrants on and off campus (P_5_) is 56, and it accounts for 10% of the total population. The high risk level, medium risk level and low risk level of express carrier infection (P_6_) were 1.25, 2.91, and 95.84%, respectively. The level of students' knowledge of the epidemic (S_1_) is 3.4. The percent of the very strong level, strong level, general level and weak level of students' awareness of risk (S_2_) were 9.16, 81.67, 5.92, and 3.25%, respectively. Cleaned up campus health dead spots, disinfected public places, equipped with disinfectants and hand sanitizer were finished for the situation of campus environment renovation (S_3_). The percent of the propaganda of anti-epidemic culture (S_4_) on the school's official website, official account, Douyin, and other platforms is 75.68%. The expert group thought that the personnel control measures (S_5_) were slightly defective. The epidemic prevention and control department asked the periodic nucleic acid testing (S_6_) is three times a week. The expert group thought that the distance between the students is ~1.2 m for campus space management (S_7_). The satisfaction of teachers and students with online teaching based on the application of digital teaching technology (S_8_) is 91.28%. The percent of the student nucleic acid testing system registration is 88.43% for the application of digital epidemic prevention technology (S_9_). The number of the health monitoring days per student in the data system (R_1_) is 14 days. The expert group thought that the university conducted emergency drills for evaluation and made improvements based on evaluation comments (R_2_). The expert group thought that the preparation of emergency plans (R_3_) was comparatively perfect. There are two parties cooperating between universities and other subjects (R_4_). Quarantine settings (R_5_) were adequate quarantine areas and staff on full-day duty. Detected factors categories or values by subsystems (Pressure, state and response), with corresponding risks and weights, are reported in Tables 3–5.

**Table 3:** Epidemic risk index collection—pressure system for university resumption.

**Subsystem**	**Pressure**					
**Indicator**	***P*_1_**	***P*_2_**	***P*_3_**	***P*_4_**	***P*_5_**	***P*_6_**
Result	Low risk	Low risk	3.32	1 ≤ *n* < 2	*x*_i_ < 5%	*x*_i_ < 5%
Assignment	1.5	1.5	3.32	3	1	1
Weight	0.15	0.23	0.19	0.22	0.12	0.09

**Table 4:** Epidemic risk index collection—status system for university resumption.

**Subsystem**	**State**								
**Indicator**	***S*_1_**	***S*_2_**	***S*_3_**	***S*_4_**	***S*_5_**	***S*_6_**	***S*_7_**	***S*_8_**	***S*_9_**
Result	8.4	Strong risk awareness	Completed 3 items	*x*_i_ ≥ 50%	Slightly flawed	3 times a week	1.5 ≤ *x_*i*_* ≤ 2	90% ≤ *x_*i*_* ≤ 95%	80% ≤ *x_*i*_* ≤ 90%
Assignment	3.4	2	1	1	2	2	2	2	3
Weight	0.06	0.09	0.16	0.05	0.12	0.17	0.15	0.11	0.09

**Table 5:** Epidemic risk index collection—response system for university resumption.

**Subsystem**	**Response**				
**Indicator**	***R*_1_**	***R*_2_**	***R*_3_**	***R*_4_**	***R*_5_**
Result	14	Carried out emergency drills and improved them	The preplan preparation was relatively perfect	Cooperated with two institutions	The isolation area was sufficient and the personnel were on duty all day
Assignment	1	1	2	2	1
Weight	0.16	0.22	0.20	0.18	0.24

After calculation, *H* = 1.880 and *V* = 1.666 were obtained, indicating that the comprehensive risk of the school's resumption was low. Therefore, risk prevention and control measures needed to be improved accordingly.

## 4. Discussions

The COVID-19 pandemic highlighted the need to multiply our efforts in epidemic prevention and control to protect public health. Since young people (often asymptomatic) are important spreader of COVID-19 (1), colleges and universities need to assess the risks involved in resuming school and make evidence-based decisions. To improve the risk index, we considered more factors compared to previous studies and described the influencing mechanisms between them through the PSR model. Finally, the disaster management perspective provided a clear picture highlighting the scale of university response. In effect, while the system vulnerabilities show where the countermeasures can be applied to be effective, the PSR model describes their impact.

### 4.1. Effective response suggested from the model

Based on our evaluation results, our study judged the risks involved in resuming school and put forward specific countermeasures for reducing the risk levels. This not only protects public health security but also has some practical implications for improving the evaluation and rational decision-making abilities of all parties. Students should apply to return to school in the system given by the school according to their requirements. They should fill in the date of their return and their mode of transportation, such as bus number and other relevant information, and they can only return after the school has provided their approval. Students are required to have health and travel codes, a 14-day health monitoring information form, and nucleic acid test proof within 48 h to enter the campus. Students are required to sign the Student Commitment to Return to School and strictly comply with the requirements of returning to campus. After entering the campus, the school will immediately disinfect students' luggage, bags, and other items, and conduct nucleic acid tests at designated locations. The school told students not to walk around the campus without special reasons and to narrow their scope of activities as far as possible. Students are not allowed to return to school without verification. Schools can hold lectures on epidemic prevention and control knowledge and relevant laws and regulations regularly, both online and offline. At the same time, information about epidemic prevention and control and national prevention and control policies should be posted on the school's publicity board and dormitory bulletin board. Because online teaching is adopted during closed-loop management, schools should actively take measures. Schools should strengthen the awareness of students and teachers regarding digital teaching technology and the use of teaching software so that they can correctly and skillfully use Dingding, Rain Classroom, Tencent Conference, and other platforms for teaching, ensuring the smooth progress of courses. Teachers should change their management mode from offline to online in a timely fashion. They should also make full use of the functions of each lecturing platform, such as check-in, links, video, and submitting homework, to innovate their teaching and enhance the effectiveness of their student management to ensure students continue with an appropriate level of learning engagement. However, the school should coordinate and improve the student management network platform system, collect all students' personal and facial information, and form a complete data management database. By doing so, the school can improve the accuracy of management, reduce the workload, and achieve high levels of management efficiency. The school should carry out refined prevention and control work in strict accordance with the relevant regulations of the national and provincial CDC, insist on regular nucleic acid testing and health reports, and strictly isolate migrants. The organization's institutional framework should also be optimized to clarify the responsibilities for epidemic prevention and control. Schools should ensure basic medical security and that they have adequate supplies, and implement real-name registration applications. School leaders and related management personnel should not only perform their respective duties but also give responsibilities to student party members. The school should organize students to be on duty every day in designated places, such as restaurants and libraries, to supervise students' daily behaviors so that disease prevention procedures can be fully implemented among students.

### 4.2. Response suggested to the case study

The results of the case study show that the university's comprehensive risk of resuming classes is low risk, and the university can allow students to return to classes in terms of epidemic prevention and control. In terms of catastrophes, the university and most of its students were at a low risk level. The size of returning students and delivery of express have little impact on the epidemic prevention and control of the university. The transportation of students back to school is the most critical factor in the disaster. This requires the establishment of rules for returning students to school. In terms of vulnerability, campus epidemic prevention equipment, epidemic prevention culture publicity, health testing, epidemic prevention drills and quarantine Settings play an active role in prevention and control. However, risk awareness among students and digital epidemic prevention are hindering epidemic control. This requires increasing students' awareness of risks and using big data intelligence to enhance epidemic prevention. Other aspects should also be effectively addressed. In general, the university should further improve and optimize the transportation mode of students returning to campus, students' risk awareness and digital epidemic prevention to enhance the epidemic prevention and control effect. Based on the above analysis, the following measures are proposed. (1) Improve students' return to school information statistics. According to the arrival time of students by plane, train (high-speed railway) and other public transportation, school buses and special buses can be arranged at the airport, high-speed railway station and other transportation stations to reduce the risk of infection on the return trip. (2) The school vigorously publicized how individuals could contribute to epidemic prevention and control, provide role models, and create a cultural atmosphere for epidemic prevention and control on campus. Schools can conduct publicity through online platforms, shoot high-quality and positive short videos on epidemic prevention and control, and regularly release and update these materials on Douyin, Kuaishou, and other platforms to expand the scope of publicity and influence. In this way, students can improve their epidemic and risk awareness and regulate their behavior in strict accordance with institutional requirements to deal with the current severe situation with the correct attitude. (3) Schools should establish an epidemic prevention command platform and control center, and formulate a 24-h duty system for epidemic prevention and control. Additionally, on-duty staff should carry out training and education to ensure timely responses to all kinds of emergencies.

### 4.3. Limitations and strengths of the study

Our study had some limitations, which deserve further study and attention. First, the functions to evaluate the risk of factors derived from subjective evaluations although they are based only on the experience of the risk assessment expert group composed of experts in various fields such as medicine, education and emergency management. Second, our study only used one case study while comparisons could help to calibrate the measure tool. The research system should be expanded according to the varying situations of different universities and more indicators should be included in the evaluation process. However, our model (to the best of authors knowledge) is to date one of the most complete describing the complex interaction mechanism of factors that affects the university spread of the virus. In addition, artificial intelligence technology could be gradually introduced to find more factors and as support of factors weights assignment.

## Data availability statement

The raw data supporting the conclusions of this article will be made available by the authors, without undue reservation.

## Ethics statement

Ethics review and approval/written informed consent was not required as per local legislation and institutional requirements.

## Author contributions

Conceptualization: SY, YW, and LM. Methodology: LM and YW. Software and writing—review and editing: LM and SY. Validation: YW and SY. Writing—original draft preparation: YW and TD. All authors contributed to manuscript revision, read, and approved the submitted version.

## Footnotes

^1^In June 2022, the following classification was introduced: Over the past 14 days, areas with no confirmed cases nor new cases are defined as low risk; areas with no more than 50 newly confirmed cases or a cumulative total of more than 50 confirmed cases and no cluster of outbreaks are defined as moderate risk. Areas with more than 50 cumulative confirmed cases and clusters of outbreaks are defined as high risk.
