# Supplementary material for: Measuring the impact of the COVID-19 epidemic on university resumption and suggestions for countermeasures
Source: Front Public Health. 2022 Dec 19;10:1037818. doi: 10.3389/fpubh.2022.1037818 (PMC9806250; doi:10.3389/fpubh.2022.1037818)
Supplement: Supplementary file 1 [file Table_1.docx]

**Supplemental material: Risk indicators of university resumption under an epidemic situation.**

| **System Name** | **Name of Index** | **Index Description/ Evaluation Method** | **Sources of Data** | **Evaluation, Grade, and Assignment of Values** | **Instruction (The risk assessment expert group must be composed of experts in various fields such as medicine, education and emergency management)** |
| --- | --- | --- | --- | --- | --- |
| **Pressure** | Epidemic risk level of students’ residence ($P_{1}$) | Indicators such as imported cases and the number of local infections were comprehensively reflected | Statistics from returning students and government websites | Low-risk zone (1.5)  Medium-risk zone (2.5)  High-risk zone (3.5) | The statistics was calculated from the regional risk level issued by the regional Health Commission.  Risk level at the place where more than 80% of the students are located. (80% can be determined by the risk assessment expert group composed of experts in various fields such as medicine, education and emergency management.) |
|  | Epidemic risk level of the school’s location ($P_{2}$) | Comprehensive reflected in the school’s imported cases, the number of infections in the area, and other indicators | Statistics from government websites | Low-risk zone (1.5)  Medium-risk zone (2.5)  High-risk zone (3.5) | Risk levels issued by the regional Health Commission |
|  | Means of transportation back to school${(P}_{3}$) | $\sum_{j=1}^{4} n_{j}\times k_{j}$ | Statistics from returning students | $n_{j}$ quantifies the risk value for each mode of transportation, $k_{j}$  is the proportion of the number of people using the mode of transportation, and the graded values were as follows:  Self-driving (1)  Taxi (2)  Train/R (3)  Other (4) | Students fill out the online shared form questionnaire. |
|  | Size of the university population $(P_{4}$) | The number (*n*) of students returning to school /1,000 | Statistics from returning students | *n*<0.5 (1)  0.5≤*n*<1 (2)  1≤*n*<2 (3)  2≤*n* (4) | Summary: Students fill out the online shared form questionnaire. |
|  | The number of migrants on and off campus${(P}_{5}$) | Number of students entering and leaving the school/total number of students in the school | Statistics of people entering and leaving the school | $\begin{matrix} X_{i}<5\%(1) \\ 5\%\leq X_{i}<15\%(2) \\ 15\%\leq X_{i}<25\%(3) \\ X_{i}\geq25\%(4) \end{matrix}$ | The college janitor fill out the online shared form questionnaire. Risk level thresholds can be determined by the risk assessment expert group composed of experts in various fields such as medicine, education and emergency management. |
|  | Express carrier infection ($P_{6}$) | The proportion of express deliveries from medium- and high-risk places out of all deliveries | Data query | $\begin{matrix} X_{i}<5\%(1) \\ 5\%\leq X_{i}<15\%(2) \\ 15\%\leq X_{i}<25\%(3) \\ X_{i}\geq25\%(4) \end{matrix}$ | Express deliveries station fill out the online shared form questionnaire. Risk level thresholds can be determined by the risk assessment expert group composed of experts in various fields such as medicine, education and emergency management. |
| **State** | Students’ knowledge of the epidemic ($S_{1}$) | Some students were randomly selected to assess their knowledge of epidemic prevention and control using a questionnaire or test | Questionnaire or online test | $\begin{matrix} X_{i}\geq8(4-3\times\frac{X_{i}-8}{10-8}) \\ X_{i}<8(4) \end{matrix}$ | Students fill out the online shared form questionnaire. The scores were calculated and averaged. |
|  | Students’ awareness of risk${(S}_{2}$) | The importance students attached to epidemic prevention and control | Observe students’ daily behavior | Very strong risk awareness (1)  Strong risk awareness (2)  General risk awareness (3)  Weak risk awareness (4) | These items include wearing masks, alcohol disinfection, etc. The monitor of each class fills out the online shared form questionnaire. (80% can be determined by the risk assessment expert group composed of experts in various fields such as medicine, education and emergency management.) |
|  | The situation of campus environment renovation ($S_{3}$) | Cleaned up campus health dead spots; disinfected public places; equipped with disinfectants and hand sanitizer | Field trips | Completed 3 items (1)  Completed 2 items (2)  Completed 1 item (3)  Not started (4) | The head of epidemic control checks these items and fills out the online shared form questionnaire. |
|  | Propaganda of anti-epidemic culture${(S}_{4}$) | Promoted prevention and control culture on the school’s official website, official account, Douyin, and other platforms; cultural lectures on fighting the epidemic were held | Background and active record data | $\begin{matrix} X_{i}\geq50\%(1) \\ 30\%\leq X_{i}<50\%(2) \\ 15\%\leq X_{i}<30\%(3) \\ X_{i}<15\%(4) \end{matrix}$ | The publicity department of the school count the number and methods of anti-epidemic activities and fills in questionnaires. Risk level thresholds can be determined by the risk assessment expert group composed of experts in various fields such as medicine, education and emergency management. |
|  | Personnel control measures ${(S}_{5}$) | The organizational framework of colleges and universities | Field investigation and management record inquiry | Met the requirements (1)  Slightly defective (2)  Major defects (3)  Failure to formulate a reasonable organizational institutional framework (4) | Whether the organizational framework of colleges and universities was reasonable; whether the personnel control measures to meet the requirements of territorial documents and national policies were formulated according to the specific conditions. Risk level can be determined by the risk assessment expert group composed of experts in various fields such as medicine, education and emergency management. |
|  | Periodic nucleic acid testing ${(S}_{6}$) | The frequency of nucleic acid testing | Personnel statistics | Once a day (1)  Three times a week (2)  Twice a week (3)  Once a week (4) | Policy requirements of the epidemic prevention and control department. |
|  | Campus space management ($S_{7}$) | We evaluated the distance between people in public areas (Unit: M) | Field trips | $\begin{matrix} X{}_{i}<1(4) \\ 1\leq X_{i}\leq1.5(3) \\ 1.5\leq X_{i}<2(2) \\ X_{i}\geq2(1) \end{matrix}$ | Students sat apart in the school canteen, library, and classroom, and we evaluated the distance between people in public areas, such as the library, classroom, and canteen (Unit: M). The head of epidemic control checks the item and fills out the online shared form questionnaire. Risk level thresholds can be determined by the risk assessment expert group composed of experts in various fields such as medicine, education and emergency management. |
|  | The application of digital teaching technology${(S}_{8}$) | The satisfaction of teachers and students with online teaching | The questionnaire survey | $\begin{matrix} X_{i}\geq95\%(1) \\ 90\%\leq X_{i}<95\%(2) \\ 80\%\leq X_{i}<90\%(3) \\ X_{i}<80\%(4) \end{matrix}$ | Students fill out the online shared form questionnaire. Risk level thresholds can be determined by the risk assessment expert group composed of experts in various fields such as medicine, education and emergency management. |
|  | The application of digital epidemic prevention technology$(S_{9}$) | Student nucleic acid testing system registration | Background data | $\begin{matrix} X_{i}\geq95\%(1) \\ 90\%\leq X_{i}<95\%(2) \\ 80\%\leq X_{i}<90\%(3) \\ X_{i}<80\%(4) \end{matrix}$ | The head of epidemic control checks the item and fills out the online shared form questionnaire. Risk level thresholds can be determined by the risk assessment expert group composed of experts in various fields such as medicine, education and emergency management. |
| **Response** | Daily health reports for students ($R_{1}$) | The number of the health monitoring days per student in the data system | The school database | $\begin{matrix} X{}_{i}\geq14(1) \\ 7\leq X_{i}\leq14(2) \\ 0\leq X_{i}<7(3) \end{matrix}$  The system was not established (4) | The database of the epidemic prevention and control department. Risk level thresholds can be determined by the risk assessment expert group composed of experts in various fields such as medicine, education and emergency management. |
|  | Emergency drill and evaluation ($R_{2}$) | Whether the school carried out epidemic drills and drill evaluations | Query the drill file report | Conducted emergency drills for evaluation and made improvements based on evaluation comments (1)  Conducted emergency drills and assessments but did not improve all assessments (2)  Conducted emergency drills but did not conduct drill evaluations (3)  Emergency drills were not conducted (4) | The head of epidemic control checks the item and fills out the online shared form questionnaire. Risk level thresholds can be determined by the risk assessment expert group composed of experts in various fields such as medicine, education and emergency management. |
|  | Preparation of emergency plans ($R_{3}$) | Prepared for all kinds of emergencies | File query | Well-prepared contingency plans for various emergencies (1)  Comparatively perfect preparation of various contingency plans (2)  Inadequate preparation of contingency plans for various emergencies (3)  Failure to prepare for various emergencies (4) | The head of epidemic control checks the item and fills out the online shared form questionnaire. Risk level thresholds can be determined by the risk assessment expert group composed of experts in various fields such as medicine, education and emergency management. |
|  | Cooperation between universities and other subjects${(R}_{4}$) | Whether colleges and universities cooperated with local governments and surrounding medical and health institutions | Field trips | Collaborated with three organizations (1)  Collaborated with two organizations (2)  Worked with an organization (3)  Failure to cooperate with organizations (4) | The head of epidemic control checks the item and fills out the online shared form questionnaire. Risk level thresholds can be determined by the risk assessment expert group composed of experts in various fields such as medicine, education and emergency management. |
|  | Quarantine settings${(R}_{5}$) | Whether the school set up enough isolation areas according to the current number of students and whether they set up all day on-duty staff | On-the-spot investigation and on-duty record inquiry | Adequate quarantine areas; staff on full-day duty (1)  Inadequate quarantine areas; staff on full-day duty (2)  Adequate quarantine areas; staff not on duty all day (3)  Inadequate quarantine areas; staff not on duty all day (4) | On-the-spot investigation and on-duty record inquiry |
